# Development of an Internet of Things–based real-time greenhouse gas and weather monitoring system for precision dairy farming

**DOI:** 10.3168/jdsc.2025-0862

**Published:** 2025-12-01

**Authors:** Keshawa Dadallage, Marina Madureira Ferreira, Alejandra Zapata-Salazar, Diego A. Ceballos, Lav R. Khot, Francisco A. Leal Yepes

**Affiliations:** 1 Department of Biological Systems Engineering, Center for Precision and Automated Agricultural Systems, Washington State University, Prosser, WA 99350; 2 Department of Population Medicine and Diagnostic Sciences, Precision Livestock Health Laboratory, Cornell University College of Veterinary Medicine, Ithaca, NY 14853

## Abstract

The environmental footprint of dairy production is one of the most pressing challenges faced by the industry globally. Our study aimed to develop and validate a cost-effective sensing solution for real-time monitoring of dairy farms' GHG emissions and microclimatic conditions. Each of the integrated sensing nodes was equipped with carbon dioxide (CO_2_), methane (CH_4_), and ammonia (NH_3_) gas sensors, along with an all-in-one weather sensor. Sensing nodes were validated against gold-standard measurements using open-circuit respiration chambers with individual cows under controlled conditions. The CH_4_ emissions (133.0 ± 22.5 ppm, mean ± SD) showed an overall correlation (r = 0.46) with the gold-standard respiration chamber (166.0 ± 32.8 ppm) across all 3 d. However, the correlation changed over time, with a strong correlation on d 1 (r = 0.62), a moderate correlation on d 2 (r = 0.35), and a weak correlation on d 3 (r = 0.11). In contrast, sensor node quantified CO_2_ emissions (905 ± 779 ppm) showed a weaker correlation (r = 0.019, 2,461 ± 346 ppm), indicating the need for further improvements to the sensing node. A wireless network of calibrated sensing nodes was deployed in 3 different locations within a dairy farm: dry cow pen (DCP), feed bunk (FB), and freestall beds (FSB) at a research dairy farm. The CH_4_ emissions were greater in the DCP (12.5 ± 6.65 ppm) compared with FB (2.80 ± 0.61 ppm) and FSB (2.34 ± 0.62 ppm). The CO_2_ emissions at the FB were greater (1,498 ± 1,020 ppm) compared with the DCP (534 ± 222 ppm) and FSB (724 ± 517 ppm). The NH_3_ emissions were highest in the FSB (4.24 ± 0.91 ppm) compared with DCP (2.93 ± 1.35 ppm) and FB (1.10 ± 0.44 ppm). The differences in GHG emissions across the different areas of the dairy farm may be influenced by ambient temperature, humidity, housing conditions, and manure management practices. Our sensing nodes may provide a low-cost, scalable sensing network that can offer a practical solution for continuous GHG monitoring.

## Highlights

• Sensing nodes showed moderate correlation with a gold-standard respiration chamber.
• The sensing network captured spatiotemporal greenhouse gas emission variations.
• Emissions may be linked to cattle density, housing, and manure management practices.
• Sensing nodes offer a cost-effective and scalable solution for emissions monitoring.

## Graphical Abstract

**Figure:**
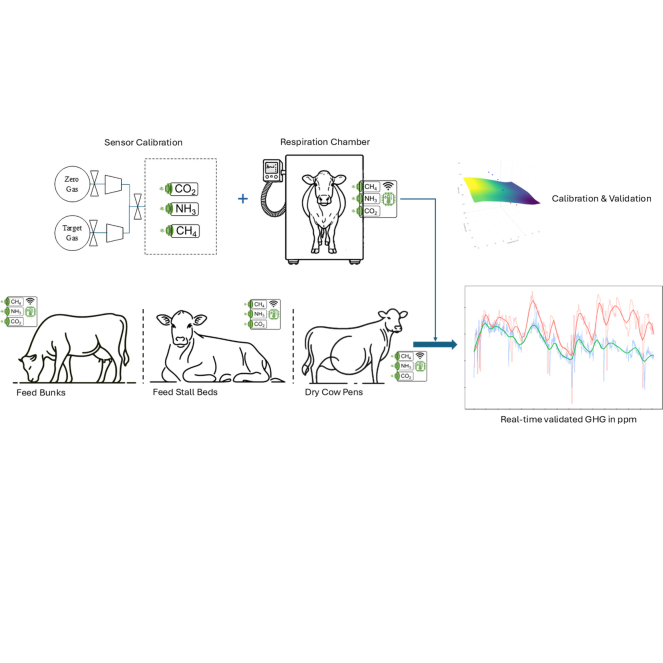
**Summary:** An Internet of Things-based wireless sensor network was developed and validated to monitor greenhouse gas emissions and environmental conditions on dairy farms. Cost-effective sensing nodes measuring methane, carbon dioxide, and ammonia were first calibrated using standardized gases and then validated against gold-standard open-circuit respiration chambers with lactating cows. The calibrated sensing nodes were subsequently deployed across different areas of a dairy farm, including feed bunks, freestall beds, and dry cow pens, to capture real-time variations in spatial and temporal greenhouse gas emissions. This scalable solution enables continuous real-time environmental monitoring. The resulting data may support precision dairy management practices aimed at assessing and mitigating the environmental footprint of dairy production systems.

The dairy industry is a vital sector of the United States agriculture, contributing greatly to the economy. However, growing consumer perceptions about the dairy industry's environmental footprint have prompted a commitment to achieve net-zero greenhouse gas emissions by 2050 (Place et al., 2022). On dairy farms, the GHG emissions include methane (CH_4_), carbon dioxide (CO_2_), and ammonia (NH_3_). Methane is a potent, short-lived climate pollutant with important global warming potential over a 20-yr time frame, making its reduction a global priority for near-term climate mitigation (IPCC, 2023). However, CH_4_ is the most important GHG produced by dairy farms. Methanogenic archaea break down feed in the cow's rumen, producing CH_4_, which is then released into the environment (Rotz et al., 2010). As CH_4_ has a much higher global warming potential than CO_2_, mitigating CH_4_ emissions is critical for the dairy industry (Garnsworthy et al., 2012). The CO_2_ emissions mainly result from cattle respiration, depending on the cow's productivity and body mass, with an average lactating dairy cow exhaling between 11 and 14.5 kg of CO_2_/d (Leytem et al., 2011). The NH_3_ emissions are primarily associated with manure handling and storage. Urinary urea undergoes enzymatic hydrolysis to form ammonium (NH_4_^+^), which can volatilize as NH_3_ under both aerobic and anaerobic conditions influenced by factors such as manure composition, temperature, and humidity (Hristov et al., 2011). Although NH_3_ is not a direct GHG, its volatilization can lead to environmental issues (Yang et al., 2021). Various methods have been employed to quantify CH_4_ and CO_2_ emissions, including radiolabeled dilution, tracheostomy, and respiration chambers, which provide comprehensive measurements but are expensive and often disrupt the natural behavior of cows (Yan et al., 2010). Commercial systems, such as the GreenFeed Emission Monitoring system (GEM, C-Lock Inc., SD), estimate enteric emissions via spot sampling but require frequent animal visits and extended measurement periods to mitigate its variability and cost limit scalability (Hammond et al., 2016).

There is a gap in affordable, scalable, and real-time GHG monitoring systems for dairy farms. Precise measurement of GHG emissions in the field is crucial for assessing mitigation efforts, verifying progress toward climate targets, and guiding data-informed decisions. Current technologies do not provide modular deployment and scalable wireless sensor network (**WSN**) capabilities suitable for facility-wide monitoring. Therefore, we hypothesized that Internet of Things (**IoT**)–enabled, low-cost sensing nodes would (1) provide GHG measurements comparable to those from open-circuit respiration chambers within ±10% under controlled conditions and (2) quantify site-specific GHG emissions and localized weather conditions across different areas of a dairy farm. Therefore, our objectives were (1) development of a robust, cost-effective, and scalable WSN to monitor GHG emissions and environmental conditions from dairy cattle operations, and (2) sensor node calibration and system validation through comparison with the gold-standard, open-circuit respiration chambers.

The WSN integrates real-time GHG emissions and weather sensing nodes. The overall architecture of the WSN (Figure 1a) integrates components designed for data acquisition, local storage, and data analytics. The schematic diagram (Figure 1b) illustrates the design of the GHG and weather sensing node. Each node uses a microcontroller unit (model: TTGO T-SIM7000G, Lilygo, Shenzhen Xinyuan Electronic Technology Co. Ltd., Shenzhen, China) to acquire data inputs from the sensor node. The system integrates 3 low-cost GHG sensors: (1) metal-oxide-semiconductor (**MOS**) CH_4_ sensor, MQ-4, range: 200 to 10,000 ppm (Hanwei Electronics, Zhongyuan Qu, China); (2) electrochemica CO_2_, MG-811, range: 350 to 10,000 ppm, (Zhengzhou Winsen Electronics Technology, HeNan, China); and (3) MOS-type NH_3_ sensor; model: MQ-135; range: 10 to 300 ppm; Hanwei Electronics, Zhongyuan Qu, China). These sensors were housed in a custom-designed 3-dimensional print made from polyethylene terephthalate glycol. An all-in-one weather sensor (ATMOS41, Meter Group Inc., WA) was integrated into the system using a serial data interface (SDI-12) protocol to measure air temperature (**T_a_**; range: −50°C to 60°C), relative humidity (**RH**; range: 0% to 100%), wind speed (**WS**; range: 0 to 30 m/s), barometric pressure (range: 50 to 110 kPa), wind gust (**WG**; range: 0 to 30 m/s), and vapor pressure (range: 0 to 47 kPa). As of the publication date, the hardware cost per GHG sensing node (excluding labor) was approximately $218.50. When integrated with a weather sensor, the total cost was approximately $2,318.50. This IoT-compatible sensing node (Figure 1c) included a Secure Digital (SD) memory card (model: SDSQUAR-016G, SanDisk Corporation, CA) for local data storage and a cellular module (model: SIM7000G, Simcom Wireless Solutions, China) providing Global System for Mobile Communications (GSM), global positioning system (GPS), and wireless data transfer capabilities. Each sensing node can be powered by two 10,050 mAh 1S3P Li-ion (model: ICR18650, Adafruit Industries LLC, NY) batteries with a battery management system (model: MCP73871, Adafruit Industries LLC, NY) to operate using either alternating current power from a wall plug or a direct current power battery connected to a 6-V solar panel (model: Voltaic-P126, Adafruit Industries LLC, NY).

**Figure 1:**
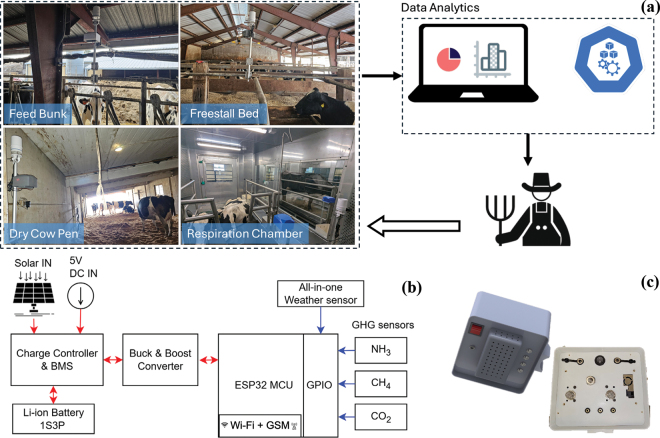
(a) Internet of Things–enabled wireless sensing network for precision dairy management through near real-time GHG and weather monitoring, (b) schematic diagram of the GHG and weather sensing node, and (c) integrated sensing node.

Each sensing node was assembled, programmed, and calibrated before its integration into the field-deployed WSN. The sensor calibration process (Figure 2a) employed a combination of zero and span calibration (Chou, 1999), and environmental compensation techniques. Zero calibration was performed using standardized calibration gases (models: J1002, F1005, H1013; MESA International Technologies, CA) to determine baseline readings in the absence of target gases (zero air, gas composition: oxygen 20.9%, total hydrocarbons <1 ppm, nitrogen ∼79.1%), compensating for inherent sensor biases. Then, span calibration was conducted to align the sensor's sensitivity with standardized gas concentrations (CH_4_: 200 and 1,000 ppm; CO_2_: 500 and 2,000 ppm; NH_3_: 10 and 300 ppm).

**Figure 2:**
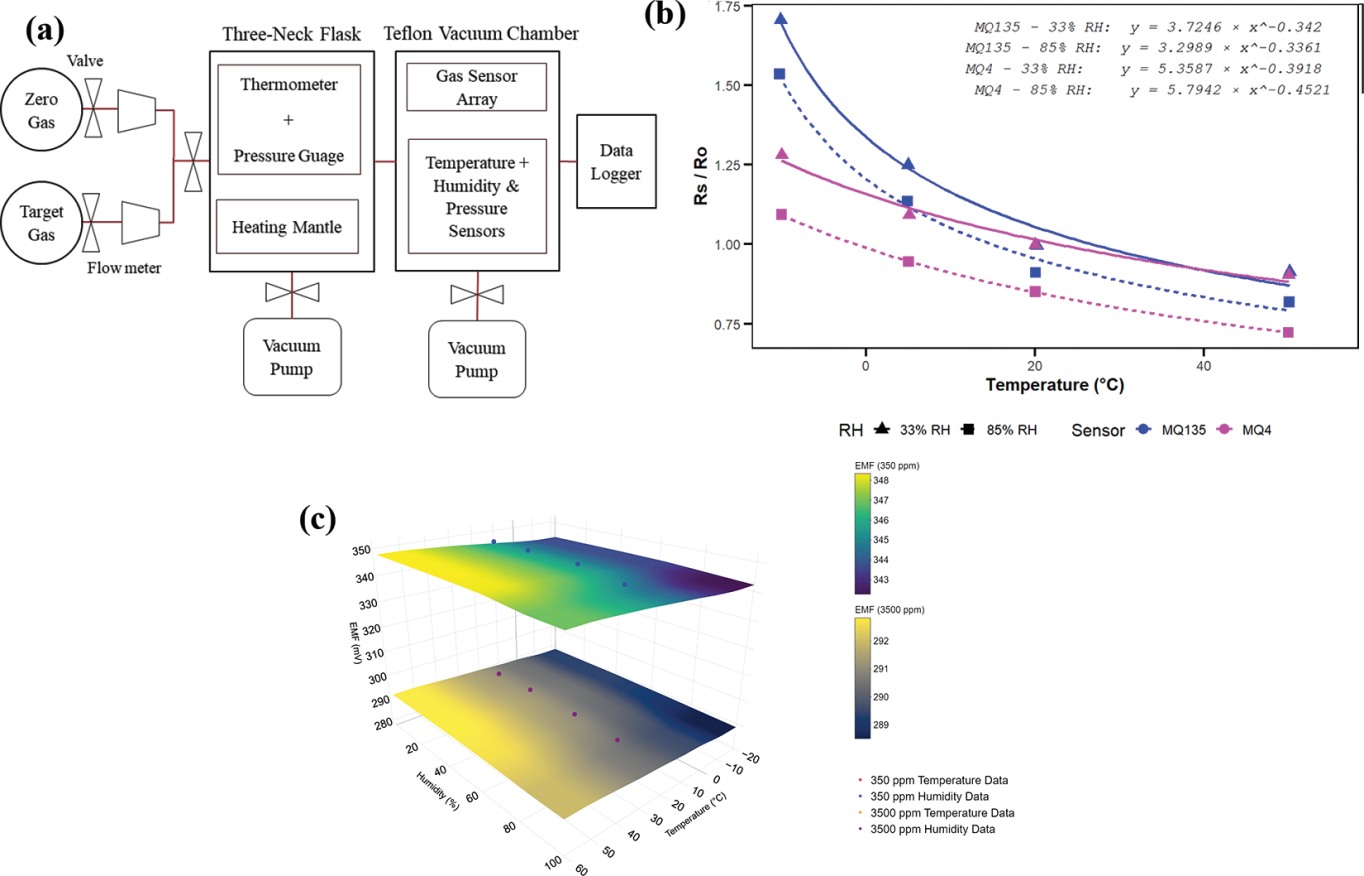
(a) Experimental setup schematic diagram for calibrating GHG sensors, (b) temperature and humidity dependence of CH_4_ and NH_3_ sensors, and (c) bilinear interpolation of temperature and humidity compensation for the CO_2_ sensor.

Calibration was performed in a controlled environment using a 1,000-mL round-bottom, 3-neck flask (model: SL000714, StonyLab Inc., NY) with a pressure gauge (model: GDD016, Uharbour, China) and thermometer (model: AO-90225-15, Cole-Parmer Inc., IL). The flask was initially evacuated to a vacuum pressure of 40 kPa to eliminate any residual gases. Subsequently, standardized clean air (zero gas) or target gases were introduced into the flask until the entire flask was filled with the introduced gas analyte. A hemispherical heating mantle (model: SL000936, StonyLab Inc., NY) was used to maintain consistent thermal conditions of the gases. Once the target temperature (20°C) was stabilized, the zero gas or target gases were transferred into a chemical-resistant 1,000 mm^3^ polytetrafluoroethylene vacuum chamber.

Before calibration, MOS sensors, MQ-4 for CH_4_ and MQ-135 for NH_3_, were powered continuously for over 24 h in clean open air to preheat and stabilize the tin dioxide (SnO_2_) sensing layer, ensuring consistent and reliable baseline performance. The sensor response is measured as the voltage (**V_L_**) across load resistor (**R_L_**), whereas the total circuit voltage (**V_C_**) is applied across R_L_ and sensing element resistance (**R_S_**). The reference sensing element resistance (**R_0_**) in clean air is derived from the sensor output voltage (**V_0_**). The ratio of R_S_/R_0_ is calculated as Equation 1. During calibration, individual sensor readings were recorded under standardized conditions (**T_a_**: 20°C and RH: 65%), capturing the noncompensated gas concentrations (ppm) using Equation 3 with CH_4_ and NH_3_ standardized gases for span calibration. The sensor(s) sensitivity characteristics were extracted from the manufacturer's datasheets using the WebPlotDigitizer tool (automeris.io, 2024). To obtain compensated concentrations, temperature and humidity dependency characteristics (Figure 2b) were derived from the datasheets. Nonlinear least squares modeling and linear interpolation techniques were used to develop compensation models estimating the ratio R_S_/R_0_ at a desired RH and T_a_ (Zhang et al., 2010). Given R_S_/R_0_ values at 2 known RH levels (RH_1_: 33% and RH_2_: 85%), compensated sensor(s) response calculated using Equation 2. Y_1_ and Y_2_ denote the ratios at RH_1_ and RH_2_, respectively, and compensated concentrations were derived using Equation 3, where the regression coefficients (NH_3_: *a* = 102.91, *b* = −2.50; CH_4_: *a* = 1,038.2, *b* = −2.75) are derived from power-law fit modeling.

[1] $$\frac{R_{S}}{R_{0}}=\frac{\left(\frac{V_{C}}{V_{L}}-1\right)}{\left(\frac{V_{C}}{V_{0}}-1\right)}$$

[2] $$\begin{array}{c} {\left(\frac{R_{S}}{R_{0}}\right)}_{\mathrm{compensated}}=\\ y_{1}+\frac{\left(\mathrm{RH}-RH_{1}\right)\left(y_{2}-y_{1}\right)}{RH_{2}-RH_{1}}\left(\begin{array}{c} y_{1\left(CH_{4}\right)}=5.36\times {T_{a}}^{-0.3917}\\ y_{2\left(CH_{4}\right)}=5.79\times {T_{a}}^{-0.452}\\ y_{1\left(NH_{3}\right)}=3.72\times {T_{a}}^{-0.342}\\ y_{2\left(NH_{3}\right)}=3.30\times {T_{a}}^{-0.336} \end{array}\right)\; \end{array}$$

[3] $$\mathrm{pp}m_{CH_{4},NH_{3}}=a\times {\left(\frac{R_{s}}{R_{0}}\right)}_{\mathrm{compensated}/\mathrm{noncompensated}}^{b}$$

The solid-electrolyte CO_2_ sensor (MG811), which operates on Nernstian principles by producing electromotive force (**EMF**) proportional to the CO_2_ concentration, requires a 1-h preheating period to stabilize its internal heater. The CO_2_ sensor was calibrated following the same procedure as the CH_4_ and NH_3_ sensor. The EMF–CO_2_ relationship was modeled using a fitted polynomial (Equation 4). Bilinear interpolation (Figure 2c) generated compensation factors for T_a_ and RH for known concentrations (350 and 3,500 ppm), and linear interpolation estimated intermediate values, ensuring accurate concentration readings under varying environmental conditions.

[4] $$\begin{array}{c} \mathrm{pp}m_{CO_{2}}=\\ a+b\left(\mathrm{EM}F_{\mathrm{compensated}}\right)+c{\left(\mathrm{EMF}\right)}_{\mathrm{compensated}}^{2}\;\left(\begin{array}{c} a=227,499.31\\ b=-1,419.68\\ c=2.22\; \end{array}\right) \end{array}$$

The calibrated sensor nodes were then deployed at 2 dairy research facilities for field evaluation. All the procedures involving animals were approved by the Institutional Animal Care and Use Committee at Washington State University (protocol #6898) and Cornell University (protocol #2024-0107). Sample size calculations were not performed for the current study. In August 2024, 3 adult Holstein dairy cows in their first lactation, average DIM of 57.7 ± 19.6, and milk yield average of 28.4 ± 2.2 kg were transported from the Teaching Dairy Barn (Ithaca, NY) to the open-circuit respiration chambers at the Large Animal Research and Teaching Unit, Cornell University (Ithaca, NY), which served as the gold standard for gas emissions measurement from cattle. Cows were allowed an acclimation period of around 72 h in the facility. On the final day of the acclimation period, cows were placed in the respiration chambers for approximately 4 h to assess their adaptation to the new environment. After the adaptation period, cows entered the chambers at 0700 h and remained in the chambers until the end of the study period. Cows were milked 3 times a day inside the chambers at 0400, 1100, and 1900 h. Cows were fed to achieve ad libitum intake, with a minimum of 5% refusals, and had access to water ad libitum. The diet formulation included the following ingredients as a percentage of the total of DM: corn silage 36.06%, haylage 15.44%, whey permeate 2.64%, molasses 1.12%, and premix grain 44.74%. Measurements from the respiration chambers were collected every 10 min, and sensing nodes were collected at 1-min intervals over 3 d. At the Knott Dairy Farm in Washington State (Pullman, WA), sensing nodes were installed at the feed bunk (**FB**) of a freestall barn, in freestall beds (**FSB**), and in a dry cow pen (**DCP**). These locations were selected to capture a range of microenvironments and management practices within the dairy facility. Measurements were collected at 1-min intervals using sensing nodes over 4 d in January 2024. All statistical analyses were conducted using R (version 4.5.1) and RStudio (v. 2025.05.1+513, Posit Software, PBC). Sensing node data included geolocation, ambient weather conditions, hardware status, CH_4_ (ppm), CO_2_ (ppm), and NH_3_ (ppm) concentrations. The measurements from the respiration chamber included CH_4_, CO_2_, ambient temperature (°C), pressure (Pa), humidity (%), and airflow (L/s) measurements. A total of 309 paired GHG measurements were included in the statistical analysis, with respiration chamber values matched to 10-min averaged values from the sensing nodes. Outlier detection was performed using the interquartile range (**IQR**) method, where values beyond the modified bounds—defined as Q1 − 3 × IQR and Q3 + 3 × IQR were classified as outliers. A total of 8 data points (CH_4_: 8; CO_2_: 0; NH_3_: 0) were identified and excluded as outliers before inferential analysis. Means, ranges, and SD were estimated for all sensor nodes and respiration chamber measurements using the Summarytools package. Pearson correlation analysis was used to assess the correlation between sensor node data, corresponding respiration chamber measurements, and the environmental conditions. The CH_4_, CO_2_, and NH_3_ were log-transformed. A linear mixed-effects model (**LMM**) assessed GHG emissions across farm areas. The model included repeated measures, a random intercept for time, and fixed effects of location. Tukey-adjusted pairwise comparisons were performed. The respiration chamber measurements showed pooled average ± SD emissions of 165.0 ± 32.3 ppm/cow per day for CH_4_ and 2,458.0 ± 342.0 ppm/cow per day for CO_2_ over the 3-d measurement period. The respiratory chamber did not quantify NH_3_. The mean ± SD sensor node measurements were 133.0 ± 22.5 ppm/cow per day for CH_4_, 1,164.0 ± 195.0 ppm/cow per day for CO_2_, and 63.4 ± 36.1 ppm/cow per day for NH_3_. The mean ± SD environmental conditions recorded inside the chamber with the sensor nodes were T_a_ of 18.2°C ± 0.15°C and RH of 54.8% ± 1.43%. A moderate positive correlation was observed between CH_4_ in the respiration chamber and the sensor node (r = 0.46, *P* < 0.0001) over 3 d. The sensor nodes CH_4_ measurements performance changed over time (Figure 3d). The CH_4_ sensor measurements and respiration chambers had a strong correlation on d 1 (r = 0.62, *P* < 0.0001), a moderate correlation on d 2 (r = 0.35, *P* < 0.0001), and a weak correlation on d 3 (r = 0.11, *P* < 0.01). In contrast, the correlation between CO_2_ measured by the sensor node and the respiration chamber was weak and nonsignificant (r = −0.10, *P* = 0.10). The NH_3_ concentration in the chambers measured by the sensor node had a negative correlation with RH (r = −0.32, *P* < 0.0001), highlighting the need for humidity compensation. The variability in sensor responses indicates possible changes over time due to environmental factors, interference from other gases, or electronic noise, emphasizing the importance of robust calibration procedures.

**Figure 3:**
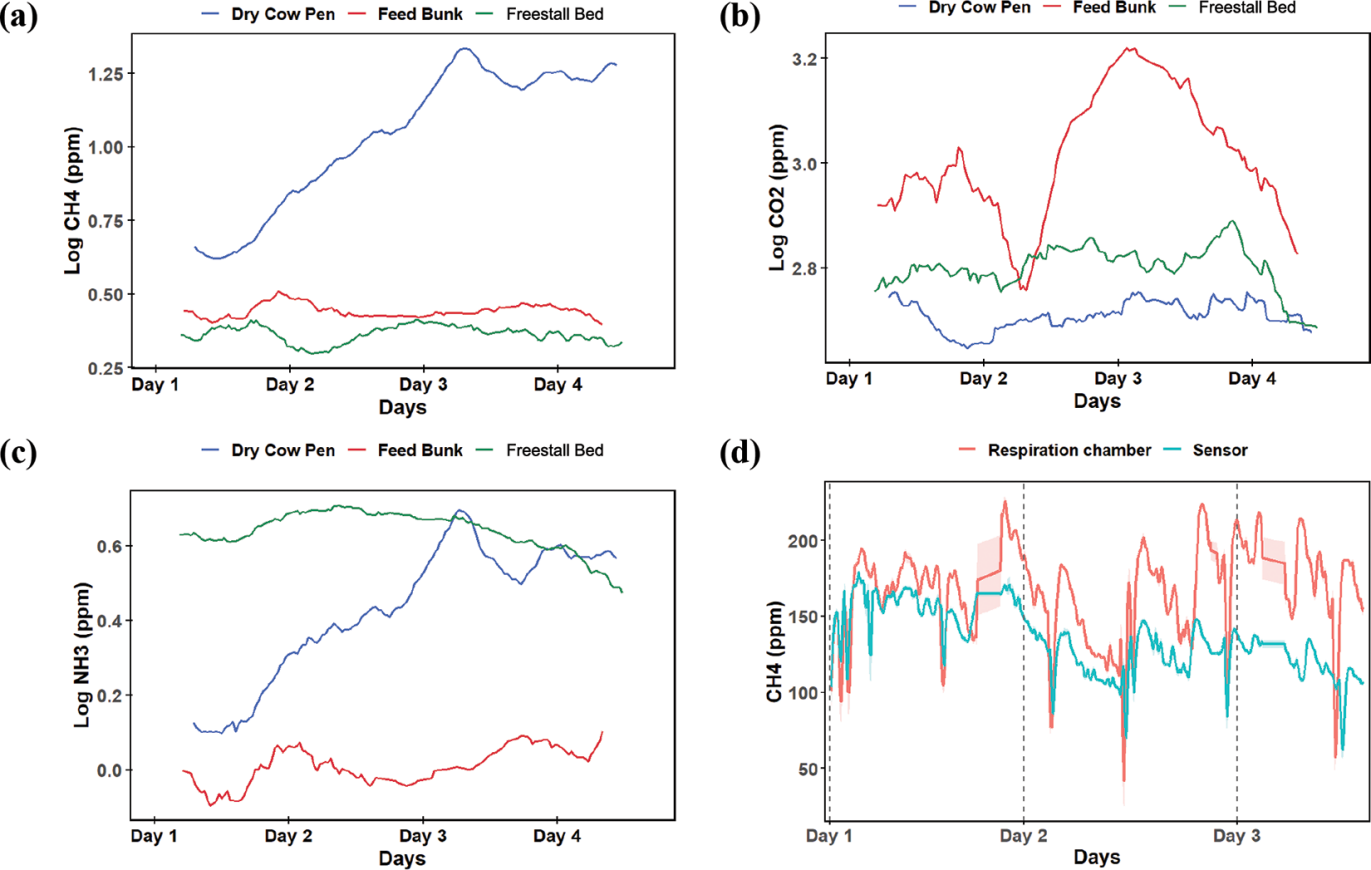
Temporal dynamics of log-transformed GHG concentrations (a) CH_4_, (b) CO_2_, and (c) NH_3_ across different cattle housing within a dairy farm, including the dry cow pen (DCP), feed bunk (FB), and freestall beds (FSB). (d) Time-series dynamics of CH_4_ emissions in the respiration chamber (gold standard) compared with integrated sensor node measurements. The shaded area represents the 95% CI.

At the dairy farm, a total of 4,681 measurements were analyzed from 2 sensors installed at each location. Using the IQR-based rule described previously, 31 outliers were identified and excluded before the statistical analysis. Overall emissions of CH_4_, NH_3_, and CO_2_ were 6.25 ± 6.30 ppm, 2.88 ± 1.59 ppm, and 727 ± 432 ppm, respectively. Environmental conditions during measurements showed an average T_a_ of 7.43°C ± 2.20°C, RH of 73.8% ± 5.22%, and WS of 0.62 ± 0.36 m/s. The LMM showed differences on GHG (CH_4_: *P* < 0.0001; CO_2_: *P* < 0.0001; NH_3_: *P* < 0.0001) across the 3 farm areas, DCP, FB, and FSB. Post hoc Tukey's honestly significant difference tests indicated that LSM CH_4_ emissions were greater in the DCP (12.5 ± 6.65 ppm) compared with FB (2.80 ± 0.61 ppm, *P* < 0.0001) and FSB (2.34 ± 0.62 ppm, *P* < 0.0001). For CO_2_ emissions, the FB was significantly elevated (1,498 ± 1,020 ppm) compared with the DCP (534 ± 222 ppm, *P* < 0.0001) and FSB (724 ± 517 ppm, *P* < 0.0001). The NH_3_ emissions were highest in the FSB (4.24 ± 0.91 ppm), with lower concentrations in DCP (2.93 ± 1.35 ppm, *P* < 0.0001) and FB (1.10 ± 0.44 ppm, *P* < 0.0001; Figure 3a–c). Environmental parameters differed across locations: T_a_ was greater at DCP (9.81°C ± 0.98°C), followed by FB (7.35°C ± 0.53°C), and lowest at FSB (4.99°C ± 0.77°C). The greatest RH was at DCP (76.4% ± 2.92%), slightly lower at FSB (75.9% ± 4.59%), and the lowest at FB (67.5% ± 2.56%). The WS was minimal at DCP (0.29 ± 0.12 m/s), increased at FB (0.68 ± 0.20 m/s), and peaked at FSB (0.92 ± 0.34 m/s). The CH_4_ showed a strong positive correlation with T_a_ (r = 0.80, *P* < 0.0001) and a moderate positive correlation with RH (r = 0.43, *P* < 0.0001), along with strong negative correlations with WS (r = −0.65, *P*  <  0.0001). The NH_3_ emissions were moderately positively correlated with RH (r = 0.65, *P* < 0.0001), weakly negatively correlated with T_a_ (r = −0.21, *P* < 0.0001), and weakly positively correlated with WS (r = 0.14, *P* = 0.0022). The CO_2_ emissions showed a weak negative correlation with both RH (r = −0.38, *P* < 0.0001) and T_a_ (r = −0.12, *P* = 0.0168), and a weak positive correlation with WS (r = 0.11, *P* = 0.0212). At the DCP, CH_4_ was strongly positively correlated with T_a_ (r = 0.87, *P* < 0.0001) and RH (r = 0.71, *P* < 0.0001), and negatively correlated with WS (r = −0.78, *P* < 0.0001). The NH_3_ showed similar patterns, positively correlating with T_a_ (r = 0.76, *P*  <  0.0001) and RH (r = 0.53, *P*  <  0.0001). No correlations were observed between CO_2_ and environmental variables in this area. At the FB, CH_4_ was weakly positively correlated with T_a_ (r = 0.21, *P* = 0.019) and showed no significant association with RH. The NH_3_ correlated weakly with T_a_ (r = 0.16, *P* = 0.069) and CO_2_ showed no significant correlations with environmental variables. At the FSB, CH_4_ exhibited no significant correlations with T_a_, RH, or WS. The NH_3_ showed a moderate positive correlation with RH (r = 0.37, *P*  <  0.0001) and a weak negative correlation with T_a_ (r = −0.17, *P* = 0.031). These findings suggest that CH_4_ emissions can be significantly correlated with temperature and WS, whereas NH_3_ emissions showed a moderate association with RH. The WSN was able to quantify and map spatial variability in gas concentrations. These results demonstrate the impact of environmental conditions and management on GHG emissions from dairy farms. Additionally, the differences across various areas of the dairy farm highlight the need for localized emissions sensing and the usefulness of the sensing network.

The sensing nodes show potential for real-time, scalable CH_4_ monitoring on dairy farms, offering a cost-effective alternative to expensive commercial systems compared with GreenFeed (approximately $80,000) and respiration chambers (costing upward of a million dollars). They can assess spatial GHG variability for precision dairy management. By integrating real-time sensing with data analytics, dairy producers can make informed decisions regarding feed composition, housing conditions, and manure management. This study has limitations including sensor drift affecting CH_4_ accuracy, weak agreement of CO_2_ measurements with respiration data, unvalidated NH_3_ sensors, and a limited sample size in controlled farm conditions. Future work should focus on long-term validation, recalibration, and scalability.
